# 

**Published:** 1964-04

**Authors:** 


					m
ts/%
April 1964
r\
: I
INDEX MEDSCUS
IjTC A^Gt :
voL-issuE. _7.2r_B.13-J
INDEXER ?j3
: .Tr-vj?ai^ - a
i
?FllSrV I
MEDICAL JOURNAL OF THE SOUTH WEST
|ol 79 (ii) NO 292

				

